# Laboratory diagnosis of von Willebrand disease in the age of the new guidelines: considerations based on geography and resources

**DOI:** 10.1016/j.rpth.2023.102143

**Published:** 2023-06-30

**Authors:** Emmanuel J. Favaloro, Leonardo Pasalic

**Affiliations:** 1 Haematology, Sydney Centres for Thrombosis and Haemostasis, Institute of Clinical Pathology and Medical Research, NSW Health Pathology, Westmead Hospital, Westmead, New South Wales, Australia; 2 School of Dentistry and Medical Sciences, Faculty of Science and Health, Charles Sturt University, Wagga Wagga, New South Wales, Australia; 3 School of Medical Sciences, Faculty of Medicine and Health, University of Sydney, Westmead Hospital, Westmead, New South Wales, Australia; 4 Westmead Clinical School, University of Sydney, Westmead, New South Wales, Australia

**Keywords:** diagnosis, exclusion, laboratory testing, von Willebrand disease, VWD, von Willebrand factor, VWF

## Abstract

von Willebrand disease (VWD) is considered the most common bleeding disorder and arises from deficiency and/or defect in the adhesive plasma protein von Willebrand factor (VWF). Diagnosis of VWD requires clinical assessment and is facilitated by laboratory testing. Several guidelines for VWD diagnosis exist, with the latest American Society of Hematology, International Society on Thrombosis and Haemostasis, National Hemophilia Foundation, and World Federation of Hemophilia 2021 guidelines presenting 11 recommendations, some of which have drawn controversy. In the current narrative review, we provide additional context around difficulties in laboratory diagnosis/exclusion/typing of VWD, with a focus on developing countries/resource-poor settings. In particular, there are many variations in assay methodology, and some methods express high assay variability and poor low-level VWF sensitivity that compromises their utility. Although we favor an initial 4-test assay panel, comprising factor (F) VIII coagulant activity, VWF antigen, VWF glycoprotein Ib binding (VWF:GPIbR or VWF:GPIbM favored over VWF Ristocetin cofactor) and VWF collagen binding, we also provide strategies for laboratories only able to incorporate an initial 3-test assay panel, as favored by the latest guidelines, to improve diagnostic accuracy.

## Essentials

• Laboratory diagnosis/exclusion of von Willebrand disease (VWD) requires laboratory assessment.
• Several guidelines provide recommendations for diagnosis of VWD.
• Developing countries are greatly challenged to diagnose VWD, struggling to adhere to guidelines.
• We provide strategies for resource-poor settings to improve laboratory diagnosis of VWD.

## 1 Introduction

von Willebrand disease (VWD) is reportedly the most common congenital bleeding disorder and may also arise as an acquired defect called acquired von Willebrand syndrome (AVWS) [1,2]. VWD and AVWS arise as a result of a deficiency of, or defect in, the adhesive plasma protein called von Willebrand factor (VWF). In addition to plasma, VWF also resides in the alpha granules of platelets and the Weibel Palade bodies of the vascular endothelium.

VWF performs several functions [3], including (i) binding to platelets—primarily via the glycoprotein Ib (GPIb) receptor, but additionally to GPIIb/IIIa (also known as integrin αIIbβ3); (ii) binding to the subendothelial matrix, primarily via the protein collagen; and (iii) binding to coagulation factor (F) VIII (FVIII) and protecting this protein from degradation. The main aim of these functions is to facilitate primary hemostasis and contribute to secondary hemostasis, thus preventing blood loss through injury. In brief, upon tissue injury, VWF binds to the damaged vessel subendothelium (via collagen) and to platelets (via GPIb), thereby anchoring platelets to sites of injury. This process leads to platelet activation, release of granule contents, including more VWF and coagulation proteins (FV and fibrinogen), and eventual platelet aggregation. VWF also delivers FVIII to the injury site, and with platelet released cargo, helps facilitate secondary hemostasis, in part conducted on the activated platelet surface, permitting conversion of fibrinogen to insoluble fibrin, and formation of stable platelet plugs.

Fundamentally, failures of VWF to bind to platelets (via GPIb), collagen, or FVIII, compromises its function, and may lead to VWD/AVWS and bleeding [1,2]. In the hemostasis laboratory, various tests are performed to assess VWF level and function (“activity”) [4]. These *in vitro* tests try to “mimic” what happens *in vivo*, albeit not exactly matching *in vivo* interactions. The aim of this narrative review is to discuss diagnosis or exclusion of VWD/AVWS from the laboratory perspective. We explain the tests used by laboratories to identify or exclude VWD, how this process is facilitated using test panels, and how reflexing to additional tests helps to classify (or type) patients with VWD. We discuss this in view of available VWD diagnosis guidelines, as well as considering geographic realities of resource-poor settings. We therefore aim to provide strategies to circumvent reliance on particular assay(s). Discussion of VWD will in general apply to AVWS.

## 2 Classification of VWD

The classification of VWD remains essentially as outlined in guidance from the International Society on Thrombosis and Haemostasis (ISTH) Scientific Standardisation Committee (SSC) on VWF/VWD [5]. There are 6 main types of VWD: (i) type 1, in which patients present with reduced levels of functionally normal VWF; this now includes type 1C, which is characterized by an increase in VWF clearance from circulation; (ii) type 2 VWD, in which patients present with “dysfunctional” VWF, with or without reduction in plasma levels of VWF, with 4 separate types: (a) type 2A VWD, reflecting reduction of high-molecular-weight multimers (HMWM) of VWF; (b) type 2B VWD, representing hyperadhesive (or gain-of-function) VWF—here, VWF may “spontaneously” bind to platelet GPIb, leading to clearance of both (HMWM) VWF and platelets from circulation (ie, lower residual VWF activity and [mild] thrombocytopenia); (c) type 2N VWD, reflecting loss of VWF FVIII binding (VWF:FVIIIB) activity (thereby, leading to lower plasma FVIII:C levels due to increased clearance); (d) type 2M VWD, reflecting loss of VWF activity not associated with loss of HMWM VWF; and finally (iii) type 3 VWD, reflecting (virtual) absence of plasma VWF. Although not really VWD, since defects lie in the platelet GPIb receptor, inclusion of “platelet type” [PT] or pseudo-VWD in discussions related to testing is helpful, since patients present phenotypically similar to those with type 2B VWD (ie, gain-of-function GPIb leads to “spontaneous” binding to normal plasma VWF, which may lead to clearance of both (HMWM) VWF and platelets from circulation (ie, lower residual VWF activity and [mild] thrombocytopenia) [6]. These gain-of-function concepts are also important for conceptually understanding newer VWF activity assays.

Recognition of different types of VWD is more than academic since patient treatment/management differs according to VWD type, of course in addition to other considerations (eg, type of planned surgery) [7,8]. In particular, desmopressin (DDAVP), a nontransfusional form of therapy, can be utilized for minor surgical cover or to prevent bleeding episodes in type 1 VWD, in particular those with mild disease. Otherwise, the main treatment for VWD is replacement of missing/defective VWF (and in some cases replacement of missing FVIII), using VWF (or FVIII) concentrates. The utility of DDAVP is limited in patients with type 2 VWD, not useful in type 3 VWD, and considered to be contraindicated in type 2B VWD. Utility of DDAVP reflects its ability to stimulate release of VWF already stored in endothelial cells, thus leading to a doubling (or more) of functionally active plasma VWF in type 1 VWD (thereby, explaining its utility here), whereas release of “dysfunctional” VWF is the consequence in type 2 VWD (thereby, explaining general lack of utility here). As no release of VWF is achieved in type 3 VWD, DDAVP is not useful in type 3 VWD. In type 2B VWD, release of gain-of-function (hyperadhesive) VWF may lead to aggravation of thrombocytopenia.

A special consideration is made in type 2N VWD. Here, the main presenting plasma “defect” is a loss of FVIII:C, but this is caused by an inability of VWF to bind and protect FVIII from degradation. Thus, DDAVP will act to release stored dysfunctional VWF, and the low relative FVIII:C will remain. Treatment of 2N VWD is primarily VWF replacement, not FVIII replacement, since providing FVIII will only increase plasma FVIII levels temporarily —infused FVIII will quickly degrade and disappear from circulation. Instead, replaced VWF will bind to and protect any FVIII produced by the patient. Therefore, 2N VWD, reflecting low FVIII:C, needs to be distinguished from hemophilia A (also reflecting low FVIII:C), so that the correct therapy is applied (VWF replacement in 2N VWD; FVIII replacement in hemophilia A) [2,7].

## 3 How Common is VWD?

This depends on how VWD is defined. Based on epidemiologic considerations, the incidence of VWD can be identified as being around 1% of the general population [1,9]. If based on laboratory testing, with “abnormal” VWF test findings flagged to infer VWD, then the incidence of VWD might be perceived as high as 2% of the general population [10]. This is because reference ranges are defined statistically, based on distribution of test results from healthy populations, either reflecting the mean ± 2× SD of a normally distributed (Gaussian) dataset or based on percentiles for non-Gaussian distributions. In either case, ranges only identify a proportion of the normal population (eg, ∼95% for mean ± 2SD) as being “normal”; a proportion of the population, including clinically asymptomatic individuals, will thus be flagged as outside this normal range (ie, “abnormal”). A proportion of this “abnormal” group will be above the range (ie, identified as “high”), and a proportion below the range (ie, “low”). If clinicians interpret low levels of VWF or VWF activity as “identifying” VWD, then the potential incidence of VWD may be perceived as being as high as 2% of the general population. Finally, if prevalence of VWD is based on patient numbers attending clinics for investigation or treatment of bleeding or bruising, as then found to have a low level of VWF and/or activity consistent with VWD, this would approximate around 1 in 10,000 of the population (or 0.01%) for developed countries [1]. However, in developing or resource-poor countries, only those with severe disease (or symptoms) would present for investigation or treatment, and prevalence may be identified as much lower than 0.01% [1].

It is also useful to compare perceived prevalence of VWD to hemophilia A, a secondary hemostasis disorder reflecting a loss of FVIII [11,12]. Again, if based on laboratory testing alone, perceived incidence would be similar to VWD, as based on test reference ranges and laboratory/clinical interpretation [10]. If based on patients attending clinics for investigation or treatment of bleeding or bruising, as then found to have low levels of FVIII activity consistent with hemophilia A, this would approximate around 1 in 5000 of the population (or 0.02%) for developed countries [12,13], with most patients being male (given that the affected gene is the X-chromosome). Again, perceived incidence of hemophilia in resource-poor countries may be less, since only those with severe disease are likely to present for investigation/treatment [13].

**Figure 1:**
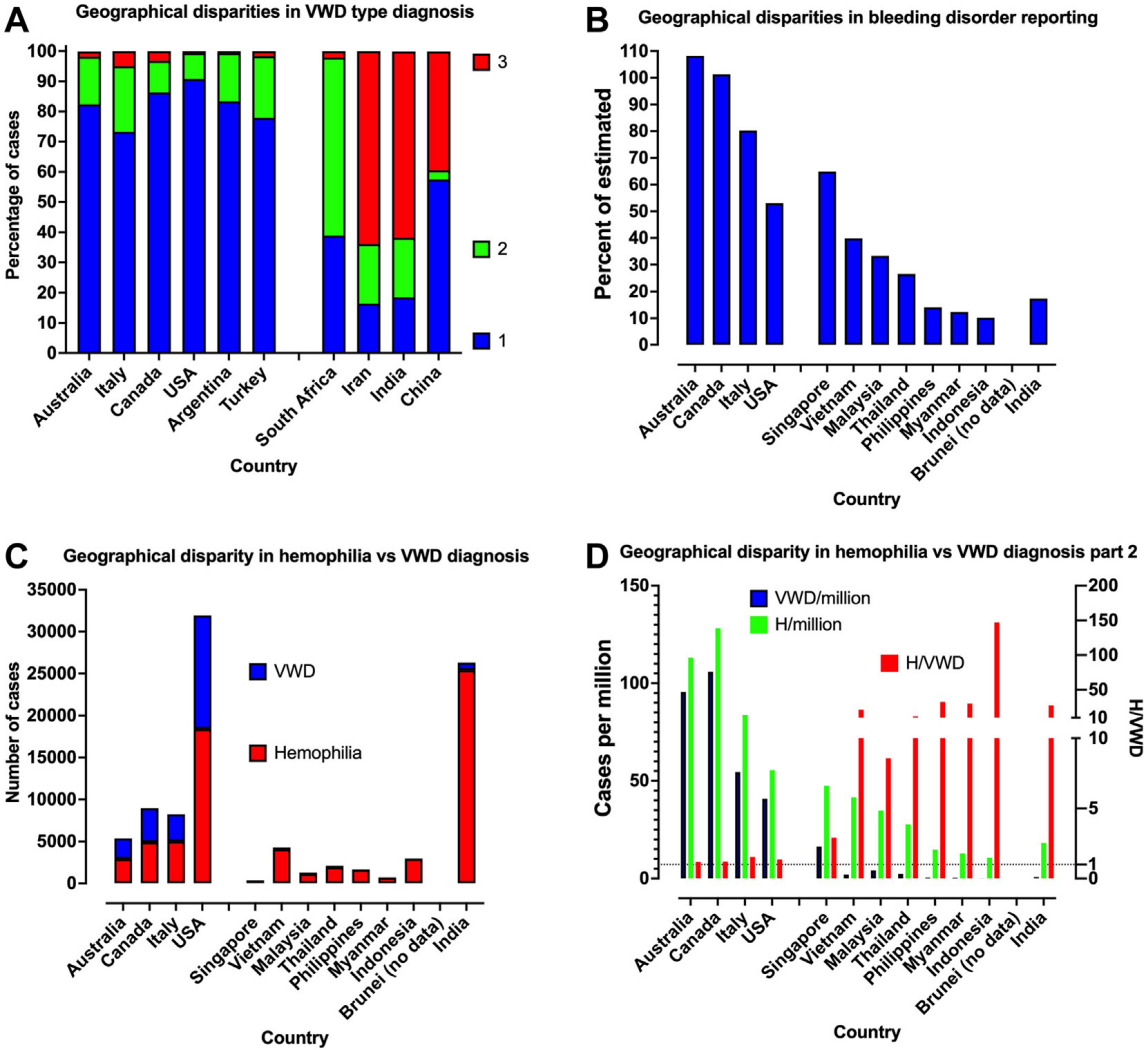
Geographic disparities in the diagnosis of bleeding disorders, including von Willebrand disease (VWD) (A) Geographic disparities in VWD type diagnosis. The distribution of VWD cases diagnosed is similar among developed countries, and primarily represented by type 1 VWD (>70% of VWD cases), then type 2 VWD (∼20% of VWD cases), followed by type 3 VWD (<5% of VWD cases). This reflects the “typical” distribution of VWD types. In developing/resource-poor countries, type 3 VWD often represents the majority of VWD cases identified, followed by type 2 and type 1 VWD cases. This probably reflects the “severity” distribution of VWD types, with only severe bleeding presentations requesting treatment. In addition, consanguinity may lead to higher incidence of type 3 in these countries. Data from reference [1]. (B) Geographic disparities in bleeding disorder reporting. In developed countries, most of the estimated burden of bleeding disorder patients are reported to the World Federation for Hemophilia (WFH), with Australia and Canada for example reporting ∼100% of the estimated number of patients with bleeding disorders. This infers that ∼100% of the estimated number of patients with bleeding disorders are being “diagnosed” in these countries. In contrast, in developing/resource-poor countries, much fewer patients than estimated are reported; this infers fewer patients with bleeding disorders are being “diagnosed” (or a relative underdiagnosis is occurring). Data from WFH Annual Global Survey 2021 (https://wfh.org/usa/research-and-data-collection/annual-global-survey/). (C) Geographic disparity in hemophilia vs VWD diagnosis. In line with the expected “similar” prevalence of hemophilia vs VWD, developed countries report similar numbers of cases of hemophilia and VWD to the WFH. In contrast, in developing/resource -poor countries, much higher numbers of persons with hemophilia are reported than with VWD. This infers a relative underreporting (or underdiagnosis) of VWD. Data from WFH Annual Global Survey 2021 (https://wfh.org/usa/research-and-data-collection/annual-global-survey/). (D) Geographic disparity in hemophilia vs VWD diagnosis part 2. Data from Figure C shown as number of cases reported to the WFH per million of population (left y-axis) for hemophilia (green bars) and VWD (blue bars), and as a ratio of hemophilia/VWD (right y-axis; red bars). In developed countries, similar numbers of cases are reported, with 50 to 100 cases per million (or ∼1/10,000 cases), and with a corresponding ratio of hemophilia/VWD approximating unity. In contrast, in developing/resource-poor countries, fewer cases of hemophilia are reported; however, much fewer cases of VWD are reported, yielding a corresponding ratio of hemophilia/VWD sometimes in excess of 100. This infers that hemophilia is underdiagnosed, and VWD grossly underdiagnosed, in developing/resource-poor countries. VWD, von Willebrand disease; WFH, World Federation for Hemophilia.

Figure 1 provides interesting perspectives on geographic disparities around diagnosis of bleeding disorders such as VWD and hemophilia. The main message is that although similar numbers of patients with VWD vs hemophilia are identified in developed countries, VWD is clearly underrecognized or underdiagnosed in certain geographic localities, in particular resource-poor countries.

## 4 Laboratory Tests Used to Diagnose VWD and Ensure Accurate Classification of VWD Type

These are summarized in Table 1. We believe it mandatory to perform ≥3 different tests as a minimal laboratory panel before VWD can be effectively diagnosed or excluded. These are the FVIII:C assay, a VWF “antigen” (VWF:Ag) assay, and a VWF GPIb binding (VWF:GPIbB) assay. For the last assay “class,” there are now 3 possible options: (a) the historical VWF activity assay, Ristocetin cofactor (VWF:RCo), or more modern alternatives of (b) ristocetin-based assays using recombinant GPIb (VWF:GPIbR), or (c) non-ristocetin based assays using gain-of-function (mutant) (recombinant) GPIb (VWF:GPIbM) [4,14,15]. Such 3-test panels are recommended by the latest American Society of Hematology, International Society on Thrombosis and Haemostasis, National Hemophilia Foundation, and World Federation of Hemophilia 2021 guidelines [15]. In our laboratory, however, and as supported by VWD diagnosis guidelines from the United Kingdom Haemophilia Doctors Organisation, as approved by the British Committee for Standards in Haematology [16], we add a fourth assay (the collagen binding (VWF:CB) assay). Subsequently, additional assays (Table 1) are required to specifically classify patients with type 2 VWD. All of these assays have limitations [4]. Some assays are particularly problematic, yielding high assay variation and poor low VWF level sensitivity [[17], [18]]. The more versions of an assay available, the more variation will be observed between sites using different versions of that assay. This is particularly true for both VWF:GPIbB and VWF:CB assays [17,19].

**Table 1:** Summary of the main tests used to diagnose/exclude von Willebrand disease.

Test	Abbreviation	What the test measures
FVIII coagulant activity	FVIII:C	The level of functional FVIII. Usually by one stage clotting assay based on a modified aPTT; sometimes by chromogenic assay (several manufacturers/suppliers).
VWF antigen	VWF:Ag	The level of VWF (both functional and not). Historically by ELISA, now mostly by latex immunoassay (several manufacturers/suppliers); sometimes by chemiluminescence (one manufacturer/supplier).
VWF glycoprotein Ib binding activity	VWF:GPIbB	Various methods (see below).
VWF Ristocetin cofactor	VWF:RCo	A VWF:GPIbB performed using platelets and ristocetin to measure platelet agglutination (several manufacturers/suppliers).
VWF GPIb binding using recombinant GPIb	VWF:GPIbR	A VWF:GPIbB performed using latex or magnetic particles, recombinant GPIb, plus ristocetin to respectively measure latex agglutination or chemiluminescence based events (one manufacturer/supplier).
VWF GPIb binding using recombinant mutated GPIb	VWF:GPIbM	A VWF:GPIbB performed using latex (commercial method; one manufacturer/supplier) or ELISA (not yet commercialized), recombinant mutated gain-of-function GPIb (but no ristocetin) to respectively measure latex agglutination or ELISA color generation.
VWF collagen binding activity	VWF:CB	Primarily performed by ELISA (a large number of manufacturers/suppliers), and increasingly by chemiluminescence (one manufacturer/supplier).
VWF FVIII binding activity	VWF:FVIIIB	Primarily performed by ELISA (one manufacturer/supplier; or using in-house/lab developed methods).
Ristocetin-induced platelet aggregation/agglutination	RIPA	Performed by platelet agglutination/aggregation (one manufacturer of ristocetin, but distributed by several suppliers).
VWF multimers	VWF:mult	Performed by agarose gel electrophoresis (one commercial semiautomated method; otherwise in-house or laboratory-developed methods).

aPTT, activated partial thromboplastin time; FVIII:C, FVIII coagulant activity; ELISA, enzyme linked immunosorbent assay; GPIb, glycoprotein Ib; VWF, von Willebrand factor; VWF:Ag, von Willebrand factor antigen; VWF:GPIbB, VWF glycoprotein Ib binding activity; VWF:RCo, VWF Ristocetin cofactor; VWF:GPIbR, VWF GPIb binding using recombinant GPIb; VWF:GPIbM, VWF GPIb binding using recombinant mutated GPIb; VWF:CB, VWF collagen binding activity; VWF:FVIIIB, VWF FVIII binding activity; RIPA, ristocetin-induced platelet aggregation or agglutination; VWF:mult, VWF multimers.

### 4.1 The basic 3-test panel for diagnosis*/*exclusion of VWD

**Figure 2:**
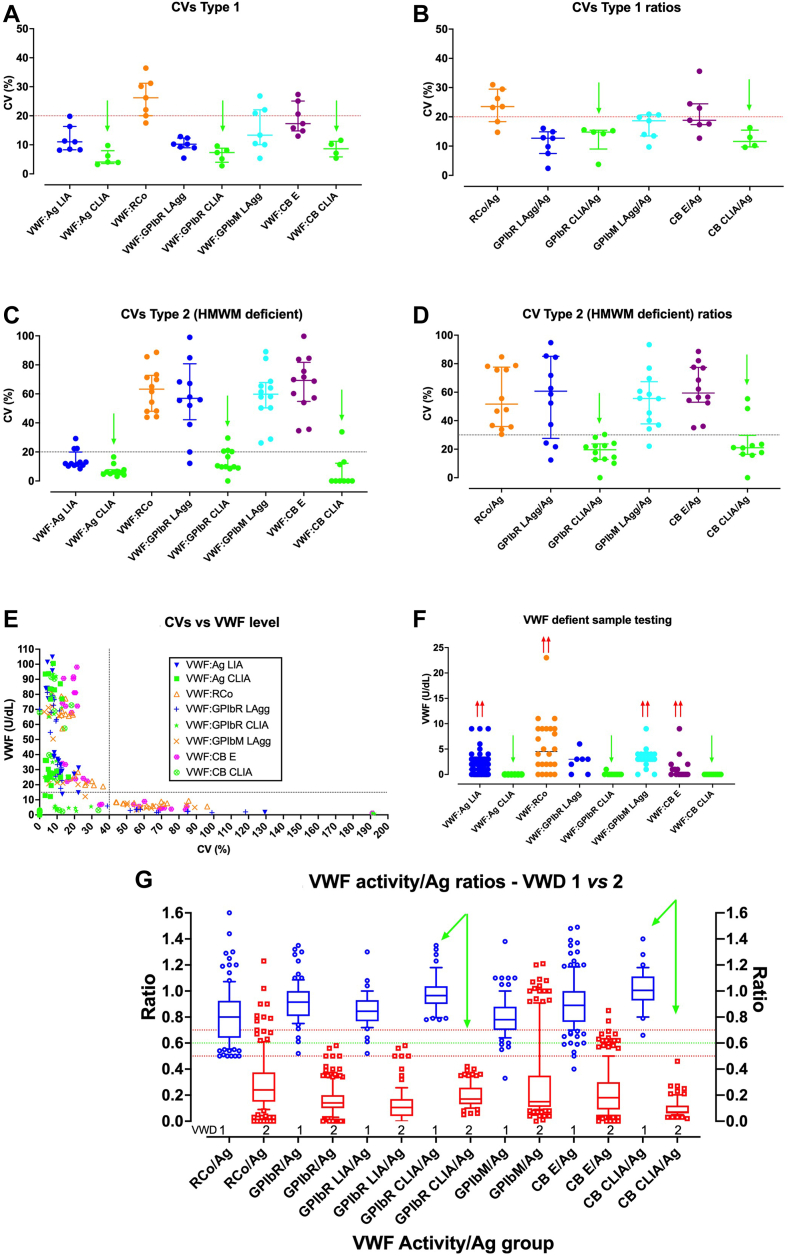
Examples of von Willebrand factor (VWF) assay variability. Coefficient of variation (CV) as a percentage, for the main VWF test types used in von Willebrand disease (VWD) diagnosis/exclusion, for samples defined as type 1 VWD (“VWF deficient”; panels A and B), or as type 2 VWD (high molecular weight [HMW] VWF deficient; panels C and D). Panel E shows main assay CVs plotted against VWF level, and panel F shows limits of VWF detection for different assays using samples devoid of VWF (ie, type 3 VWD). Panel G shows VWF activity/Ag ratios for the main assay types separated according to type 1 (VWF deficient) vs type 2 (HMW VWF deficient) samples. The main tests being: VWF:Ag (antigen), VWF:RCo (Ristocetin cofactor), VWF:GPIbR (glycoprotein Ib [GPIb] binding using recombinant GPIb), VWF:GPIbM (GPIb binding using recombinant mutated GPIb), and VWF:CB (collagen binding), as performed by latex immunoassay (LIA; VWF:Ag), platelet agglutination (VWF:RCo), latex agglutination (LAgg; VWF:GPIbR; VWF:GPIbM), chemiluminescence (immuno)assay (CLIA; VWF:Ag, VWF:GPIbR, VWF:CB) or ELISA (E; VWF:CB). In general: (i) for the separate tests, the lowest CVs are observed using VWF:Ag, and for separate methodologies using CLIA; (ii) high CVs for assays (panels A, C) translates to high CVs for assay ratios (panels B, D); (iii) CVs are lower for type 1 VWD testing than type 2 VWD testing; this is partially related to the level of VWF as shown in panel E; thus, in type 2 HMWM VWF deficient VWD, VWF:Ag levels tend to be above 20U/dL, but VWF activity levels often fall below 20U/dL; (iv) CLIA methodology shows best overall test performance. Data from the Royal College of Pathologists of Australasia Quality Assurance Program; figures modified from data previously published [18,19]. CLIA, chemiluminescence (immuno)assay; CV, coefficient of variation; HMWM, high molecular weight multimers.
  (ii) VWF:GPIbR assays represent more modern alternatives to VWF:RCo, primarily performed either by latex agglutination (automated hemostasis analyzer) or chemiluminescence (AcuStar instrument) [4,14,19,22]. In VWF:GPIbR assays, the platelets otherwise used in VWF:RCo assays are replaced with either latex (agglutination assay) or magnetic particles (chemiluminescence assay), and native GPIb (present on platelets for VWF:RCo) replaced with recombinant GPIb as attached to either latex or magnetic particles. VWF:GPIbR assays, being performed on automated platforms, should have lower assay variability and better low VWF level sensitivity than VWF:RCo assays, historically performed on platelet aggregometers, and thus should provide more robust technologies for VWD diagnosis/type assignment. In theory, VWF:GPIbR assays might also be affected by VWF polymorphisms affecting ristocetin binding in VWF:RCo, but several studies show this is not the case (at least for some polymorphisms) [23,24]. In particular, Boender et al. [24] showed similar findings for VWF:GPIbR (assessed by chemiluminescence on AcuStar) and VWF:GPIbM (using the commercial assay on Sysmex CS-5100 analyzer) in 47 patients with the polymorphism p.Asp1472His, with reported values higher than those of VWF:RCo (also performed on the CS-5100). Why the chemiluminescence VWF:GPIbR assay (marketed by the manufacturer as a VWF:RCo) is less sensitive to these polymorphisms than classical VWF:RCo is not known. However, these assays utilize different reagents and are performed in different ways. In classical VWF:RCo, washed, fixed and/or lyophilized platelets containing native GPIb are mixed with patient plasma in the presence of ristocetin. The ristocetin alters the confirmation of VWF to permit binding to platelet GPIbR, causing agglutination. Instead, the AcuStar VWF:GPIbR (“VWF:RCo”) assay is a 2-step assay in which platelets are replaced by magnetic beads and native GPIb is replaced with a recombinant fragment of GPIb (GPIbR); the GPIbR is immobilized to the magnetic particles by means of a specific monoclonal antibody that orientates the GPIbR in such a way that it can interact with the sample VWF. There is no agglutination; the reaction measure is chemiluminescence. Although we favour use of VWF:GPIbR over VWF:RCo, for resource-poor settings, VWF:GPIbR has the potential disadvantage of being less well-established, less accessible, and perhaps more expensive than classical VWF:RCo.
  (iii) VWF:GPIbM assays can be performed using either a commercial assay (by latex agglutination) or by enzyme linked immunosorbent assay (ELISA; provided reagents are available) [4,25,26]. In VWF:GPIbM assays, platelets otherwise used in VWF:RCo are replaced with latex (commercial assay) or an ELISA well plate, and native GPIb (in VWF:RCo) replaced with gain-of-function (“mutated”) recombinant GPIb. These gain-of-function assays do not require ristocetin to facilitate VWF to GPIb binding, and so VWF:GPIbM assays are *not* ristocetin-based assays. Being performed on automated analyzers, the commercial VWF:GPIbM assay should enable reduced assay variation and better low VWF level sensitivity, compared with VWF:RCo. Not being dependent on ristocetin, the VWF:GPIbM assay should also be unaffected by VWF polymorphisms otherwise affecting VWF ristocetin binding, and thus less likely (compared to VWF:RCo, and potentially to VWF:GPIbR) to generate an associated false diagnosis of (type 2A or 2M) VWD in those individuals. Again, the findings of Boender et al. [24] support this with a large number of patients with the polymorphism p.Asp1472His.^.^ On the other hand, some VWF:GPIbM assays, reflecting gain of GPIb function assays, may yield falsely high values in patients with type 2B VWD (reflecting gain-of-function VWF) [27]. Additionally, for resource-poor settings, VWF:GPIbM has the potential disadvantage of being less well-established, less accessible, and perhaps more expensive than the classical VWF:RCo.

(a) FVIII:C is a mandatory test for VWD since VWF normally binds to and protects FVIII; thus, low levels of VWF are associated with low levels of FVIII [4]. Moreover, low levels of FVIII combined with low levels of VWF compounds bleeding risk (representing defects in both primary and secondary hemostasis). Notably, FVIII is additionally lowered in type 2N VWD. However, FVIII:C testing cannot be used in isolation; a normal level of FVIII does not always exclude VWD (FVIII:C levels will be normal in many patients with “mild” type 1 VWD and also in some patients with type 2B and 2M VWD), and an abnormal FVIII:C does not always establish a diagnosis of VWD (hemophilia A is actually more likely). So, additional tests for VWF level and activity are mandatory.
(b) VWF:Ag is a mandatory test for VWD since it quantifies the level of VWF present in plasma [4]. The lower the VWF level, the greater the bleeding risk. However, VWF:Ag identifies both functional and non-functional VWF forms. Thus, VWF:Ag cannot be used in isolation since normal VWF:Ag levels do not always exclude VWD (levels will be normal in some type 2B and 2M VWD patients), and abnormal VWF:Ag levels, although potentially consistent with VWD, do not identify VWD type (type 1 or type 2 VWD?). Thus, testing for VWF activity is also required.
(c) VWF:GPIbB assays are also mandatory tests for VWD diagnosis/exclusion since they provide markers for a major VWF activity, being binding of VWF to its platelet receptor (GPIb) [4]. The main question here is which VWF:GPIbB assay to use—VWF:RCo, VWF:GPIbR, or VWF:GPIbM? There are advantages and limitations for each.
  (i) The main limitations of VWF:RCo, reflecting platelet agglutination assays, especially in historical sense, is their high assay variability and poor low-level VWF sensitivity (Figure 2) [17,19]. These limitations compromise VWD detection and accurate VWD type designation, leading to false diagnosis of type 1 VWD as type 2 VWD and *vice versa*, as well as poor discrimination of type 3 VWD. Falsely low VWF:RCo values may also arise with certain VWF polymorphisms affecting ristocetin binding, with the possibility of false VWD diagnosis and incorrect type 2A or 2M VWD assignment (depending on other tests also performed) [20,21]. However, for resource-poor settings, VWF:RCo has the advantage of being well-established and is likely the cheapest of the VWF:GPIbB assays to perform.

### 4.2 The extended 4-test panel

**Figure 3:**
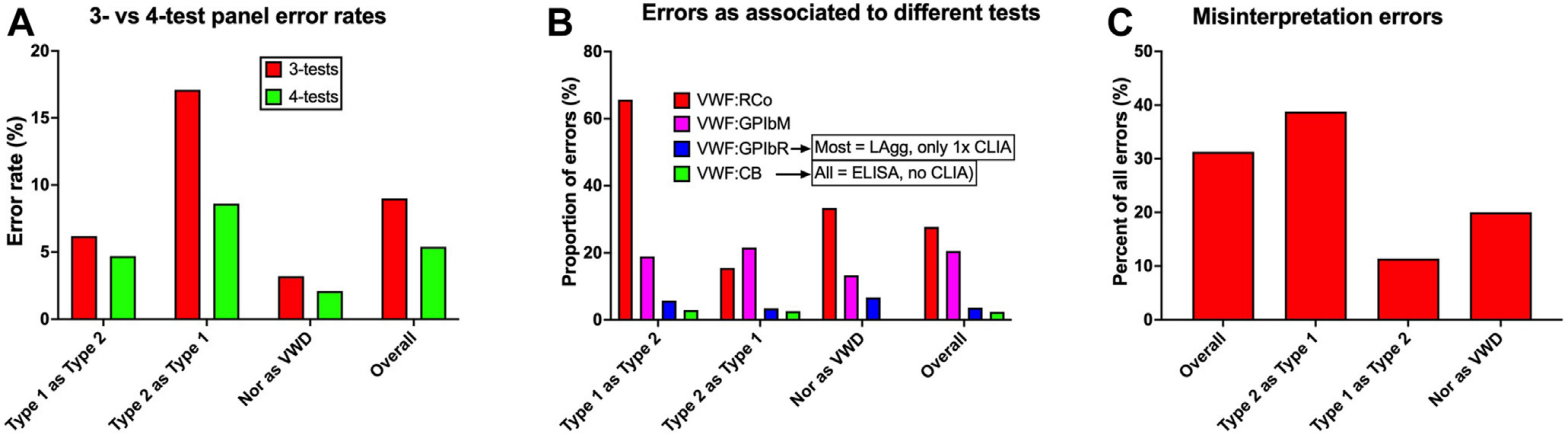
Errors in laboratory von Willebrand disease (VWD) diagnosis. (A) Identification errors according to 3- or 4-test panels used for investigation of VWD; 3-test panel = FVIII:C, VWF:Ag, and VWF:GPIb binding assays (VWF:RCo, VWF:GPIbR, or VWF:GPIbM); 4-test panel = 3-test panel plus VWF:CB. (B) Errors associated to different test types. Shown is the proportion of errors as associated to each test type for different errors (ie, type 1 VWD [VWF deficient] incorrectly reported as type 2 [high molecular weight multimer VWF deficient] or *vice versa*; normal samples incorrectly reported as VWD; overall errors). VWF:RCo testing was associated with the most errors, followed by VWF:GPIbM. VWF:GPIbR and VWF:CB use were associated with very few errors; most errors associated with VWF:GPIbR were with the latex agglutination method (just a single error using CLIA methodology), and all errors associated with VWF:CB were with ELISA methodology (no errors using CLIA methodology). (C) Misinterpretation errors as a percentage of all reporting errors: This figure identifies errors associated with misinterpretation of the laboratories own test panel results. Error types as per panel C, thus, (i) where test results were consistent with type 2 VWD, but the laboratory reported the case as type 1 VWD; (ii) where test results were consistent with type 1 VWD, but the laboratory reported the case as type 2 VWD; (iii) where test results were consistent with testing of a normal sample, but the laboratory reported the case as VWD; (iv) overall misinterpretation errors. Data from the Royal College of Pathologists of Australasia Quality Assurance Program; figures modified from data previously published [18,19].

This simply adds a fourth test, the VWF:CB, to the basic 3-test panel above. This reflects our own longstanding VWD testing practice [28], as supported by the United Kingdom guidelines on VWD diagnosis [16]. Briefly, collagen binding represents a different, but still major, VWF activity additional to platelet GPIb binding, including the *in vivo* correlate mentioned earlier. Thus, defects in collagen binding increase the bleeding risk above that of a defect in GPIb binding alone. Notably, patients with type 2A VWD, reflecting loss of HMWM VWF plus possible defects in VWF activity (according to genetic variant may express both defective GPIb and collagen binding), and are reported to have a more severe bleeding phenotype than patients with 2M VWD (who often reflect a loss of GPIb binding, but potentially normal collagen binding, and who do not suffer from loss of HMW VWF) [[29], [30], [31], [32], [33]]. Moreover, when directly compared in surveys of test practice, 3-test panels are associated with “laboratory VWD diagnosis” error rates approximately 2-fold higher than those performing 4-test panels (Figure 3) [17,19]. This is partly because the fourth test, being the VWF:CB, provides additional diagnostic information, which helps overcome some of the inherent limitations of VWF:GPIbB assays when used alone as the VWF activity assay. For example, laboratories that only perform 3-test panels will never accurately diagnose/discriminate 2A from 2M VWD, and will also have difficulty accurately discriminating type 1 vs type 2 VWD, especially if high assay variability of VWF:GPIbB assays translates to false functional discordance in type 1 VWD, or false functional concordance in type 2 VWD (Figure 2) [17,19]. The role of the VWF:CB in helping to distinguish 2A and 2M VWD is detailed elsewhere [28,[30], [31], [32], [33]], and summarized in the next section.

### 4.3 Use of assay ratios

Incorporation of test ratios provides context around specific VWF activity for various assay markers [4]. The main assay ratios are as follows:

(a) FVIII:C/VWF:Ag ratio provides context around the specific activity of FVIII:C, relative to the level of VWF. In healthy individuals and in those with types 1, 2A, 2B, 2M, and 3 VWD, levels of FVIII:C are relatively concordant (often a little higher) compared with those having VWF, so ratios approximate unity (or >0.7; note: do not try to calculate ratios in type 3 VWD, as the denominator will be very close to 0). This ratio is low in hemophilia A and type 2N VWD. Alternatively, a low ratio may potential represent a preanalytical issue—see section on Preanalytical Issues in VWD Diagnosis/Exclusion.
(b) VWF:GPIbB/Ag ratio provides context around the specific VWF activity of platelet GPIb binding, and refers to any of VWF:RCo/Ag, VWF:GPIbR/Ag, or VWF:GPIbM/Ag. These ratios will be normal (ie, >0.7) in healthy individuals and in those with types 1 and 2N VWD. Ratios are not calculated in type 3 VWD, but levels would be expected to be concordant (noting, however, that some assays cannot detect levels of VWF to <5 or <10 U/dL; Figure 2F). These ratios will expectedly be low in types 2A, 2B, and most cases of 2M VWD. The low ratio in these cases is either due to low relative HMWM VWF (2A and 2B VWD) and/or presence of *VWF* variants expressing defective GPIb binding (2A or 2M VWD).
(c) VWF:CB/Ag ratio provides context around the specific VWF activity of collagen binding, and will be normal (ie, >0.7) in healthy individuals and in those with types 1 and 2N VWD. Ratios are not calculated in type 3 VWD but levels would be expected to be concordant (noting again limits of assay detection; Figure 2F). The ratio will expectedly be low in type 2A, 2B, and some cases of 2M VWD. The low ratio in these cases is either due to low relative HMWM VWF (2A and 2B VWD) and/or *VWF* variants expressing defective collagen binding (a proportion of type 2A and 2M VWD).

The ability of the VWF:CB to add value to that of the VWF:GPIbB assays is thus manyfold. First, given high assay variability for most VWF:GPIB assays (Figure 2) [17,19], there is a possibility of both false functional discordance in type 1 VWD and false functional concordance in type 2 VWD. The VWF:CB thus acts as a kind of control; ie, discordance of both VWF:GPIbB/Ag and VWF:CB/Ag probably points to a loss of HMWM, although a type 2M VWD is possible. However, discordance of only one of VWF:GPIbB/Ag or VWF:CB/Ag is not compatible with loss of HMWM (assuming assays are suitably sensitive to HMWM) and therefore points to either type 2M VWD or potentially false discordance or concordance for one assay type (usually, this is VWF:GPIbB). Here, repeat testing for confirmation is critical; should repeat testing confirm the pattern, then type 2M VWD is likely. However, the ability for VWF:CB assays to help discriminate 2A and 2M VWD depends on the VWF:CB assay performed. ELISA based VWF:CB assays, mostly based on use of collagen type I and/or III, are usually normal in type 2M VWD, as they appear to be insensitive to most of these *VWF* variants. In contrast, the chemiluminescence VWF:CB assay, based on a collagen type III peptide fragment, tends to be sensitive to these variants, and thus, although useful for initial identification of type 2A and 2M cases, are less useful for 2A vs 2M discrimination [22,28,[30], [31], [32], [33]].

### 4.4 The extended test repertoire and assignment of VWD type

An initial test panel, be it a 3- or 4-test panel, remains the initial step in the diagnostic journey, being mostly used for the provisional identification/exclusion of VWD. Thus, initial panels identify the presence or absence of VWD, but further testing (Table 1) is required to correctly classify patients, especially for type 2 VWD, which is important to ensure optimal clinical management.

(i) All initial test results normal: VWD/AVWS can largely be excluded (∼90% confidence level). This is especially true if VWF levels are >100 U/dL [34,35]. Two caveats prevent 100% confidence for exclusion: (a) there are some rare cases of VWD, notably 2M VWD, that can only be identified by performing VWF:CB assays, and so if only a 3-test panel is performed, these cases will be missed [32,33]; (b) VWF is an acute phase protein, which increases following exercise, anxiety, infection, and during pregnancy. Thus, normal VWF levels occasionally mask (mild) type 1 VWD. The strategy, in patients with relevant clinical history, is repeat testing performed on fresh samples following exclusion of these issues (ie, no exercise, anxiety, infection, pregnancy). Anxiety/stess related increases in VWF is particularly relevant in pediatric patients.
(ii) All initial VWF test results are low, but concordant (ie, VWF activity/Ag ratios are normal or >0.7): the patient may have type 1 VWD, but again with caveats. First, having low levels of VWF *per se* (ie, below normal reference range cutoff) does not in itself define VWD—clinical evidence of bleeding/bruising must also be present. The latest VWD diagnosis guidelines [15] recommend assigning type 1 VWD to individuals with VWF levels <30 U/dL, with concordant VWF activity/Ag ratio, and to those with VWF levels between 30 and 50 U/dL, concordant VWF activity/Ag ratio, and appropriate clinical history. Individuals with VWF levels between 30 and 50 U/dL, with concordant VWF activity/Ag ratio, but without appropriate clinical history, can be assigned to a group of “low VWF as a risk factor for bleeding,” but not to VWD status. Another reason type 1 VWD may be diagnosed erroneously in individuals with low, but concordant, VWF levels is because of false concordance due to assay variability (Figure 2) [17,19]. This is particularly problematic with classical VWF:RCo, but can also occur with VWF:GPIbR and VWF:GPIbM. It can also represent a problem with ELISA based VWF:CB assays. The main recommendation is to repeat VWF tests for confirmation, using fresh samples, to ensure the same pattern is observed. Importantly, the risk of false concordance in type 2 VWD when using both classes of activity assay (ie, VWF:GPIbB and VWF:CB) at the same testing occasion is low, further justifying use of an initial 4-test panel.
(iii) All VWF test results are very low (ie, <5U/dL) but concordant: the patient may have type 3 VWD. However, be warned that some tests have very poor low-level VWF sensitivity and cannot accurately identify levels of VWF <10 U/dL (Figure 2E). We recommend repeat testing on fresh samples for confirmation, ensuring that tests accurately detect VWF levels <5U/dL before identifying patients as type 3 VWD.
(iv) a low FVIII:C/VWF:Ag is observed (ie, <0.7): the patient may have hemophilia A or 2N VWD, or the low FVIII:C reflects a preanalytical issue (eg, delayed, inappropriate transport; Table 2). We recommend repeat testing on fresh samples for confirmation, ensuring appropriate collection, storage, and transportation of samples. If test findings are confirmed, hemophilia A and 2N VWD can be differentiated using either a VWF:FVIIIB assay or by genetic analysis of *F8* and *VWF* genes, respectively [15,36].

**Table 2:** Preanalytical issues affecting the accurate diagnosis of von Willebrand disease (VWD).

Condition or event	Effect on VWD diagnosis	Comments/strategies
Underfilled blood collection tubes, or pooling of underfilled tubes.	Citrate anticoagulant dilution effect; can lead to false type 1 VWD.	Education of collection and medical staff.
Clotted blood collection or serum.	Preferential entrapment of high-molecular-weight multimers of VWF (HMWM VWF) that may lead to false type 2A or 2B pattern.	Education of collection and medical staff.
Delayed transport or high temperatures during transport.	Loss of FVIII (highly labile); can lead to false type 2N VWD or hemophilia A pattern.	Education of transport teams.
Transport of whole blood refrigerated or on ice.	Activation of platelets and FVII, leading to potential absorption of HMWM VWF; can lead to false type 2A or 2B pattern.	Education of transport teams.
Plasma filtration (historical process for sample preparation for lupus anticoagulant [LA]). If for investigation of prolonged aPTT, LA may be co-ordered with VWF assays.	Can lead to adhesion of HMWM VWF onto filter.	Education of sample processing team; double centrifugation is now the recommended process for LA sample processing.
Ineffective mixing of plasma post freezing/thawing.	Can lead to “gradients” in the sample; depending on where the sample is drawn for testing, may yield false type 1, type 2A or 2B pattern.	Education of test laboratory staff to mix samples well post freezing/thawing using several inversions prior to testing.
Exercise/stress/infection/pregnancy.	VWF and FVIII are acute phase proteins, and levels rise in these situations; can mask a type 1 VWD.	Exclude these events/repeat testing when these events can be excluded.
The choices we make: a. What a clinician orders is what they get. b. What tests or test panels a laboratory chooses. c. What guidelines we follow.	a. if only factor assays are ordered, hemophilia A may be diagnosed instead of VWD (since VWF testing not performed). b. high rates of VWD misdiagnosis if too limited a test panel or “bad” tests are employed. c. different guidelines may differ in recommendations and may yield different diagnostic outcomes.	a. Education of clinicians to request VWF tests in addition to FVIII, if clinically indicated. b. Laboratories should employ the best tests available to them, and use a minimal 3-test panel (and preferably 4-test panel if possible). c. Follow evidence-based guidelines as much as possible, and employ additional strategies for VWD diagnosis/exclusion if guidelines cannot be followed due to test unavailability.
The choices made for us: a. What a clinician orders is what gets done. b. The type and range of tests available for our instruments. c. The type and range of tests available, as approved by regulatory authorities.	a. if only factor assays are ordered, hemophilia A may be diagnosed instead of VWD (since VWF testing not performed). b. high rates of VWD misdiagnosis if too limited a test panel or “bad” tests are employed. c. Some geographies have restrictive regulatory policies that yield limited test availability that compromises accurate VWD diagnosis.	a. Education of clinicians to request VWF tests in addition to FVIII if clinically indicated. b. Laboratories should employ the best tests available to them, and otherwise be aware of the limitations in their own tests and test panel. c. additional strategies for VWD diagnosis/exclusion may be required for accurate VWD diagnosis.

aPTT, activated partial thromboplastin time; FVII, factor VII; FVIII, factor VIII; HMWM, high molecular weight multimers (of VWF); VWD, von Willebrand disease; VWF, von Willebrand factor. Summarized from [39].
(v) the VWF:GPIbB/Ag (ie, VWF:RCo/Ag, VWF:GPIbR/Ag, or VWF:GPIbM/Ag) ratio is low or discordant (ie, <0.7): type 2A, 2B, or 2M is possible (as is PT-VWD). Caveats here include false discordance due to assay variability, with VWF:RCo being most problematic. We recommend repeat testing on fresh samples for confirmation. Again, a 4-test panel is better since the risk of a false discordance using both separate assay classes (ie, VWF:GPIbB and VWF:CB) at the same time is low. If discordance is confirmed, then patients should be further evaluated to ensure correct VWD typing. We perform ristocetin-induced platelet agglutination (RIPA) first, as this will help identify or exclude 2B or PT VWD (both show response to low ristocetin concentrations) [32,37]. If type 2B or PT VWD is identified, these can be distinguished using RIPA mixing assays or genetic analysis of *VWF* and platelet *GP1b* genes, respectively [15,37]. If type 2B and PT VWD are excluded (RIPA response only with high concentrations of ristocetin), then options are type 2A or 2M VWD. These can be distinguished using VWF multimer analysis, or sometimes using VWF:CB ELISA assays [15,32,33]. Type 2A VWD will show significant loss of HMWM VWF, whereas type 2M VWD will not. For type 2M VWD, the most common forms express dysfunctional GPIb binding, but normal collagen binding (thus, low VWF:GPIbB/Ag but normal VWF:CB/Ag). Note however, that most type 2M cases will also show low VWF:CB/Ag ratios if using the chemiluminescence assay and that some cases of type 2M will show low VWF:GPIbB/Ag and low VWF:CB/Ag using ELISA assays [[30], [31], [32], [33]]. We therefore always recommend genetic analysis of *VWF* for all cases yielding consistently low VWF:GPIbB/Ag and/or VWF:CB/Ag ratios unless the defect is related to AVWS. Notably, AVWS can be considered as a diagnosis when there is new onset of bleeding with no preceding history of bleeding with challenge, in the setting of cardiac valve disease, monoclonal gammopathy or myelo/lymphoproliferative disorders [2].
(vi) the VWF:CB/Ag ratio is low or discordant (ie, <0.7): like the situation for VWF:GPIbB/Ag, type 2A, 2B, 2M or PT VWD is possible. Caveats here include false discordance due to assay variability, this being problematic with some ELISA assays (Figure 2) [17,19]. We recommend repeat testing on a fresh sample for confirmation. If confirmed, patients should be further evaluated to ensure correct VWD typing, essentially as outlined above for low VWF:GPIbB/Ag, starting with RIPA, progressing to RIPA mixing (to identify or exclude 2B or PT VWD), multimer analysis and/or genetic testing [15,32,38].
(vii) Both VWF:GPIbB/Ag and VWF:CB/Ag are low: most likely scenarios are type 2A or 2B or PT VWD (but 2M VWD remains possible). If only one of VWF:GPIbB/Ag and VWF:CB/Ag is low (and confirmed upon repeat), then this is inconsistent with loss of HMWM VWF, and so type 2A or 2B or PT VWD is unlikely, and instead 2M VWD is likely. If VWF:GPIbB/Ag is consistently low but VWF:CB/Ag is consistently normal, we would identify these patients as type 2M_GPIb_. If VWF:GPIbB/Ag is consistently normal but VWF:CB/Ag is consistently low, we would identify these patients as type 2M_CB_ [32].

## 5 Preanalytical Issues in VWD Diagnosis/Exclusion

Laboratories perform sample testing and do not “diagnose” VWD on their own, which involves clinical judgment. If accurate testing is performed, then laboratories can accurately define VWF and FVIII levels and activity present in patient samples assessed, but this does not necessarily reflect accurate representation of patients, since sample quality may have been compromised. A correct diagnosis of VWD requires appropriate sample collection and processing. Many preanalytical issues can compromise VWD diagnosis (Table 2) [39]. We also highlight less well-recognized “preanalytical” issues of “the choices we make” and “the choices made for us,” particularly relevant in resource-poor settings as follows:

a) Clinical requests: what clinicians order is what laboratories perform. If clinicians only order factor assays, and low FVIII:C is identified, then hemophilia may be incorrectly diagnosed in a patient with VWD, since VWF testing was not performed. This is particularly problematic for type 3 VWD, where levels of FVIII:C are generally <10U/dL, with presenting patient symptoms potentially “hemophilia-like,” but can also arise with other VWD types. Thus, education of clinicians is critical, and VWF testing should be performed in patients with appropriate clinical histories. In some cases, reflex VWF testing may be appropriate (eg, FVIII:C level <10U/dL on first patient presentation).
b) The tests and test panels chosen by laboratories or chosen for them: laboratories should select the best tests and test panels available since these selections will influence their ability to correctly identify or exclude VWD. Sometimes laboratories are forced to use particular tests or test panels (eg, due to prior tender arrangements, predominance of certain suppliers in a region, test costs, or local regulatory limitations); in such cases, laboratories should be aware of limitations of tests they employ and communicate these to clinicians as well as develop strategies that mitigate arising problems in VWD diagnosis.
c) The guidelines that are followed: laboratories should follow the latest evidence-based guidelines as much as possible, or else justify any deviations from that guidance.

## 6 VWD Diagnosis Guidelines

Table 3 provides a synopsis of the most recent VWD diagnosis guidelines, and certain variations related to laboratory testing. Historical context is important, with recommendations to perform VWF:RCo assays in older guidelines “modernized” in latest VWD guidelines to preferential testing with either VWF:GPIbR or VWF:GPIbM (given the theoretically lower assay variability and better low VWF level sensitivity), with VWF:GPIbM “favored” since this should not yield false low values in patients with *VWF* mutations affecting ristocetin binding [15]. However, we have raised issues with this “suggestion,” based on a low grade of evidence, since VWF:GPIbR may also be less affected than VWF:RCo [23,24]. Moreover, in local experience, VWF:GPIbR expresses lower assay variability and better low VWF level sensitivity than VWF:GPIbM [18,19,40]. Another “suggestion” [15] (Supplementary Table 1) is to use a cutoff value of 0.7 instead of 0.5 for VWF activity/Ag ratios to distinguish type 1 and 2 VWD, since a cutoff value of 0.5 may miss some type 2 VWD cases. Although we agree 0.7 is preferred over 0.5, we instead use a cutoff value of 0.6, which works better with our own instrumentation (AcuStar) and VWF methods (based on chemiluminescence)—indeed, this cutoff (0.6) for this combination permitted complete separation of type 1 and 2 VWD cases assessed by participants of our local external quality assurance program, and outperforms any cutoffs using other methods/combinations (Figure 2) [19,39,40]. The cutoff value of 0.6 is also recommended by the United Kingdom guidelines [16]. Finally, we utilize an initial 4-test panel instead of a 3-test panel for diagnosis/exclusion of VWD, again in line with the United Kingdom guidelines [16] but not supported by the latest guidelines [15]. It is clear to us that use of an initial 4-test panel provides for increased assurance around diagnostic accuracy and is also associated with fewer diagnostic errors [17,19].

**Table 3:** A synopsis of differences and similarities in various guidelines.

Reference	Group	Initial test panel no.	Test panel types
Sadler et al. [5]	ISTH SSC Classification	Not specified	VWF:RCo (and/or VWF:CB), VWF:Ag, and FVIII
Nichols et al. [41]	NHLBI (USA)	3^a^	VWF:Ag, VWF:RCo, FVIII
Laffan et al. [16]	UK HCDO BCSH	4	VWF:Ag, VWF:RCo, VWF:CB, FVIII
James et al. [15]	ASH ISTH NHF WFH	3^a^	VWF:Ag, VWF:GPIbB∗∗, FVIII (∗∗VWF:GPIbM preferred over VWF:GPIbR preferred over VWF:RCo)

ASH ISTH NHF WFH, American Society of Hematology, International Society on Thrombosis and Haemostasis, National Hemophilia Foundation, and World Federation of Hemophilia; ISTH SSC, International Society on Thrombosis and Haemostasis Scientific Standardisation Committee; NHLBI, National Heart Lung and Blood Institute; UK HCDO BCSH, United Kingdom Haemophilia Centre Doctors' Organisation as approved by the British Committee for Standards in Haematology.

FVIII, factor VIIII; VWF, von Willebrand factor; VWF:Ag, VWF antigen; VWF:CB, VWF collagen binding; VWF:GPIbB, VWF GPIb binding; VWF:GPIbM, VWF GPIb binding using recombinant mutated GPIb; VWF:GPIbR, VWF GPIb binding using recombinant GPIb; VWF:RCo, VWF Ristocetin cofactor.

a Excludes VWF:CB in initial test panel.

Several other recommendations or suggestions from the latest guidelines have generated additional controversy, as outlined in various publications [[40], [42], [43], [44], [45]]. Some of the guideline writing group have since expanded their guidance [[46], [47], [48]]. Although these provide additional context, the systematic review pathway focuses on studies that match inclusion criteria (eg, cohort studies, cross-sectional studies) and completely omits experience from the real world of diagnostic testing captured by external quality assessment surveys [17,19,39,40,[49], [50], [51], [52], [53], [54]].

## 7 Additional considerations for Resource-Poor Settings

It should be clear from our discourse that accurate identification and diagnosis of VWD is particularly problematic in resource-poor countries (Figure 1). This may be due to several factors as follows: laboratories in these countries are more likely to perform inexpensive (in-house or laboratory-developed) tests, smaller test panels (in our experience via the Royal College of Pathologists of Australasia Quality Assurance Program, some laboratories only perform 2-test panels of FVIII:C and VWF:Ag; these laboratories will never be able to accurately diagnose/exclude VWD), may not use the VWF:CB, nor have available multimer or genetic testing [16,17]. In these locations, some additional strategies may be required to permit effective diagnosis/exclusion of VWD (Table 4).

**Table 4:** Special considerations for resource-poor countries.

Consideration	Comment/additional strategy
Laboratories have reduced access to (commercial) diagnostic tests and methods.	Use the best tests available to you; otherwise, be very aware of your lab tests/test panel strengths and limitations.
Tests/test panels likely tied to existing instrumentation/supplier limitations.	Three-test panels are subject to ∼2x error rate of 4-test panels (respectively, ∼10% vs ∼5% overall).
More likely to perform 3-test panel (typically, FVIII:C, VWF:Ag, VWF:RCo) than 4-test panel (added VWF:CB).	Repeat tests for confirmation using a fresh blood sample, and if results discrepant, repeat on a third sample.
Test costs and limited expertise likely to be major barriers to provision of diagnostic services.	Ensure staff are educated in correct interpretation of test results.
FVIII:C testing often performed in isolation (ie, without VWF test panel).	Assist partner clinicians to request the right tests for the right patient.
	Ensure assays have good low VWF level sensitivity (<5 U/dL of VWF).
	Enroll in a good external quality assessment VWF program.
	Check validity of the activity/Ag cutoff value for assays you perform; is 0.7 best? 0.7 will capture more type 2 VWD cases, but also some type 1 VWD cases, so need a strategy to undo potential misdiagnosis (eg, repeat testing, multimer analysis).
Sometimes only perform 2-test panel (FVIII:C and VWF:Ag; or VWF:Ag and VWF:RCo; or FVIII:C and VWF:RCo).	Such 2-test panels are insufficient for accurate diagnosis or exclusion of VWD, and will lead to a high rate of VWD misdiagnosis.
More likely to perform VWF:RCo than VWF:GPIbR or VWF:GPIbM.	VWF:RCo assays are still acceptable, but may have greater variability and poorer low VWF level sensitivity, so be aware of assay limitations and repeat tests for confirmation.
May not have access to VWF:CB assays, multimer analysis or genetic testing	Type 2M VWD cannot be accurately diagnosed without VWF:CB and/or multimer analysis. Type 2N VWD cannot be accurately diagnosed without VWF:FVIIIB and/or genetic analysis. Type 2B/PT-VWD requires RIPA/RIPA mixing, and/or genetic analysis. If these tests are not available in your lab, send specific samples to partner labs capable of offering those tests when required and if possible. Otherwise inform requesting clinicians of the diagnostic limitations of your tests/test panels.
More likely to be able to perform RIPA assays than genetic tests.	RIPA assays remain acceptable, but make sure that these assays are performed and interpreted correctly [37].

FVIII, factor VIIII; RIPA, ristocetin-induced platelet aggregation or agglutination; VWD, von Willebrand disease; VWF, von Willebrand factor; VWF:Ag, VWF antigen; VWF:CB, VWF collagen binding; VWF:GPIbB, VWF GPIb binding; VWF:GPIbM, VWF GPIb binding using recombinant mutated GPIb; VWF:GPIbR, VWF GPIb binding using recombinant GPIb; VWF:RCo, VWF Ristocetin cofactor.

## 8 Conclusion

**Figure 4:**
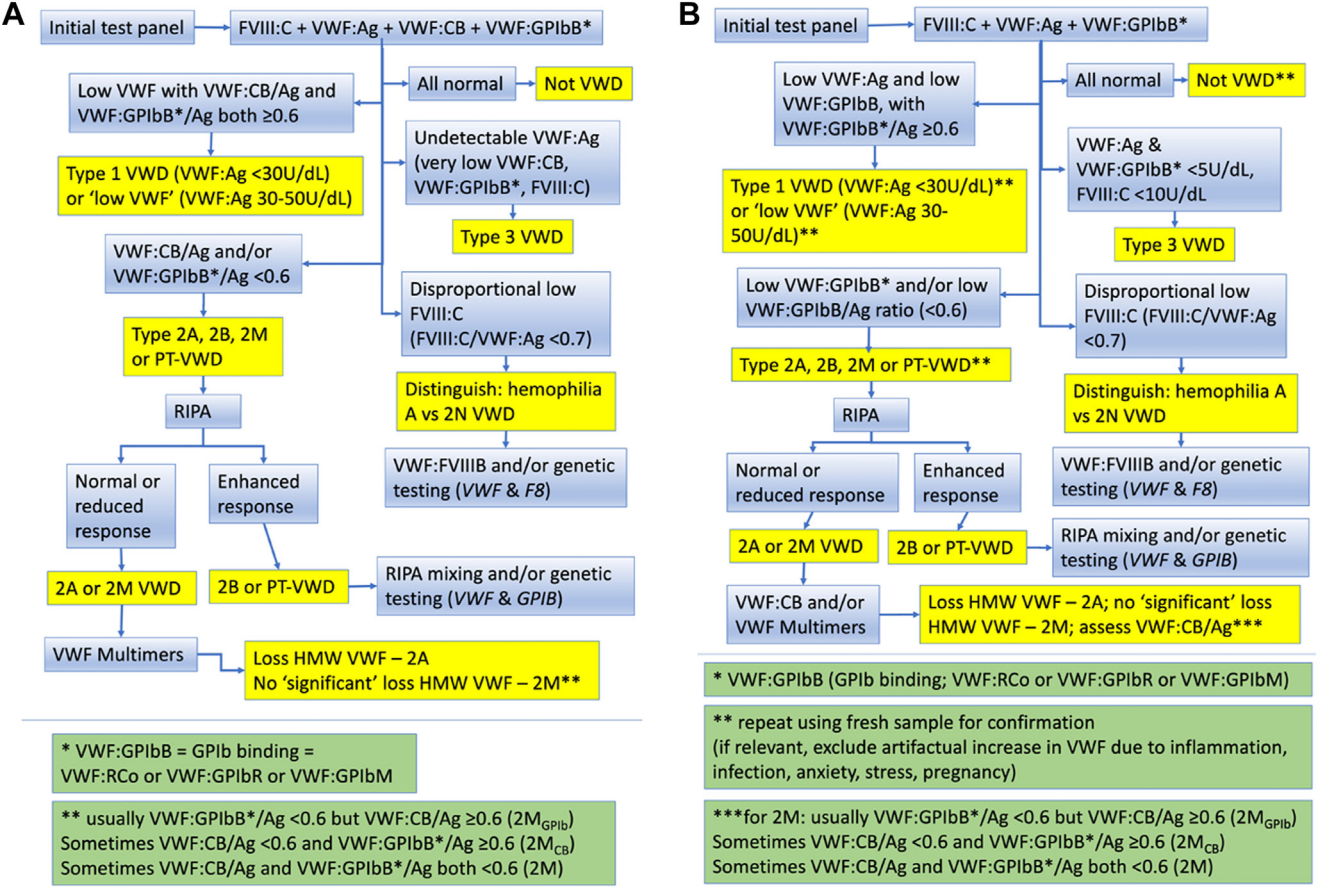
Algorithmic approaches to von Willebrand disease (VWD) diagnosis or exclusion. (A) The approach taken in our laboratory using an initial 4-test panel. (B) An alternative approach where laboratories are restricted to use of an initial 3-test panel.

In this narrative review, we provide suggestions to improve accuracy in laboratory diagnosis of VWD, in part reflecting on geographic aspects and resource-poor countries. We also provide our own algorithmic approach to the diagnosis of VWD, as based on an initial 4-test panel, as well as a potential alternative algorithm based on the more standard initial 3-test panel (Figure 4).
