# Supplementary material for: Laboratory diagnosis of von Willebrand disease in the age of the new guidelines: considerations based on geography and resources
Source: Res Pract Thromb Haemost. 2023 Jun 30;7(5):102143. doi: 10.1016/j.rpth.2023.102143 (PMC10439443; doi:10.1016/j.rpth.2023.102143)
Supplement: Supplementary Table 1 [file mmc1.docx]

**Supplementary Table 1.** Recommendations and suggestions from the latest VWD diagnostic guidelines

| **Recommendations/suggestions** | **Comments** |
| --- | --- |
| 1-3: relate to use of bleeding assessment tools (BAT) or scores | Clinical assessments are outside the control of the laboratory, but we agree these have value and may be useful in VWD diagnosis/exclusion. |
| 5: relates to previously confirmed type 1 VWD who now have VWF levels that have normalized with age  6. relates to VWF level cut-off (0.30 IU/mL vs 0.50 IU/mL) for diagnosis of VWD | Clinical assessments are outside the control of the laboratory; refer to arising controversy.^40,41^ |
| 4: suggests newer assays that measure the platelet binding activity of VWF (e.g., VWF:GPIbM, VWF:GPIbR) over VWF:RCo assay (automated or nonautomated) for VWD diagnosis, with VWF:GPIbM potentially preferred over VWF:GPIbR | Suggests = low grade of evidence  Labs should optimize assays available to them. VWF:GPIbM may not be available or easily employed in all labs. In our geography, the VWF:GPIbR assay (chemiluminescence) outperforms the commercial VWF:GPIM assay.^18,37,38^ |
| 7. suggests against using the VWFpp/VWF:Ag ratio for type 1C diagnosis, instead preferring DDAVP trials | Most labs do not perform the VWFpp test. We also prefer DDAVP trials, since this is more clinically useful, and more likely to be performed. |
| 8. suggests against a platelet-dependent VWF activity/VWF:Ag ratio cut-off of <0.5, and rather using a higher cut-off of <0.7 to confirm type 2 VWD (2A, 2B, or 2M) | Suggests = low grade of evidence  Each lab should validate cut-off in use. We also prefer a ratio of 0.7 ahead a ratio of 0.5. However, in our lab, we have validated a ratio of 0.6 as the best cut-off for our methodology. Refer to arising controversy.^38^ |
| 9. suggests either VWF multimer analysis or VWF:CB/VWF:Ag ratio to diagnose type 2 VWD for patients suspected of type 2A, 2B, or 2M | Good recommendation with which we agree. |
| 10. suggests targeted genetic testing over low-dose RIPA to diagnose type 2B VWD for patients suspected of type 2A or 2B in need of additional testing | Suggests = low grade of evidence  Genetic analysis often not available, and sometimes not clearly informative (variant may be present, but link to pathogenesis or particular VWD type assignment may be unclear). Refer to arising controversy.^39^ We prefer initial evaluation with RIPA/RIPA mixing^34^ prior to genetic testing. |
| 11. suggests using either VWF:FVIIIB or targeted genetic testing (when available) for suspected type 2N VWD | Good recommendation with which we agree. |
